# BAFF- and APRIL-targeted therapy in systemic autoimmune diseases

**DOI:** 10.1186/s41232-016-0015-4

**Published:** 2016-07-21

**Authors:** Shingo Nakayamada, Yoshiya Tanaka

**Affiliations:** The First Department of Internal Medicine, School of Medicine, University of Occupational and Environmental Health, 1-1 Iseigaoka, Yahata-nishi, Kitakyushu, 807-8555 Japan

**Keywords:** BAFF, APRIL, B cells, Tfh cells, Autoimmune diseases

## Abstract

B cells play a pivotal role in autoimmunity not only by producing pathogenic autoantibodies but also by modulating immune responses via the production of cytokines and chemokines. The B cell-activating factor/a proliferation-inducing ligand (BAFF/APRIL) system promotes B cell survival and differentiation and thus plays a prominent role in the pathogenesis of autoimmune diseases. Currently, BAFF and APRIL inhibitors are in clinical trials for systemic lupus erythematosus with significant efficacy. However, several studies have demonstrated the efficacy of the BAFF/APRIL blockade which showed considerable variability in the response to B cell-targeted therapy. This may indicate substantial heterogeneity in the pathogenesis of autoimmune diseases. Therefore, objective markers that can predict the effect of BAFF/APRIL-blocking agents could be valuable to the precision medicine linked clinically and to cost-effective therapy.

## Background

Systemic autoimmune diseases are pathologically characterized by immune complexes consisting of antigens, the activation of dendritic cells and autoreactive T cells, and the overproduction of autoantibodies secreted from activated B cells, which cause severe inflammation in various organs [1]. Although the survival of patients with autoimmune diseases has improved over the past 50 years with conventional treatments such as immunosuppressants and corticosteroids, these drugs are limited by inefficacy and intolerance in some patients. Since several autoimmune diseases such as systemic lupus erythematosus (SLE) and ANCA-associated vasculitis (AAV) remain an important cause of mortality and morbidity, innovative therapeutic approaches need to be developed.

B cells play a pivotal role in the pathogenesis of autoimmune diseases not only by producing pathogenic autoantibodies but also by modulating immune responses via production of cytokines and chemokines [2]. The potential efficacy of B cell depletion therapy has been reported in several autoimmune diseases. Rituximab, a chimeric anti-CD20 antibody, eliminates CD20-expressing pre-B and mature B cells through antibody- and complement-dependent cytotoxic activities [3]. In Japan, rituximab is approved for clinical use in childhood refractory nephrotic syndrome and AAV such as granulomatosis with polyangiitis (GPA) and microscopic polyangiitis (MPA). Despite expectations, large randomized controlled clinical trials of rituximab for non-renal and renal SLE (EXPLORER and LUNAR, respectively) did not achieve the primary goal [4, 5]. In addition, adverse reactions such as hepatitis B virus reactivation, opportunistic infections, malignancies, and inefficacy in AAV patients who were treated with rituximab have been reported in a Japanese cohort (RiCRAV) [6].

Currently, the TNF family ligands, B cell-activating factor (BAFF), a proliferation-inducing ligand (APRIL), and those receptors (BAFF receptor (BAFF-R), transmembrane activator and calcium modulator and cytophilin ligand interactor (TACI), B cell maturation antigen (BCMA), and proteoglycans) are found to play a prominent role in the pathogenesis of and are known as the potential therapeutic target for autoimmune diseases. In this review, we highlight the recent advance in the BAFF/APRIL-targeted therapy in systemic autoimmune diseases.

## Pathological significance of the interaction between B cells and Tfh cells

Disturbances of T cell and B cell functions are involved in the development of autoimmune diseases [2, 7–11]. Activated B cells function as potent antigen-presenting cells and activate autoreactive T cells. The expression of co-stimulatory molecules, such as CD40 and CD80, is enhanced on B cells in autoimmune diseases such as SLE and is involved in the interactive activation with surrounding immunocompetent cells including autoreactive T cells [8, 9]. In addition, RNA- or DNA-containing autoantigens co-ligate B cell receptors (BCRs) and Toll-like receptor (TLR)-7/9, leading to robust activation, proliferation, and differentiation of autoreactive B cells [12]. In SLE, autoantibodies produced by autoreactive B cells form immune complexes that deposit in tissues, leading to persistent inflammation and organ damage. Furthermore, it is well known that the number of memory B cells and plasmablasts correlate with disease activity in SLE [13–15]. We reported previously that the proportions of CD19^+^IgD^−^CD27^+^ class-switched memory B cells and CD19^+^IgD^−^CD27^−^ effector memory B cells tended to be higher in the peripheral blood of refractory SLE patients than in that of the control [16–18]. In contrast, B regulatory (Breg) cells, which produce interleukin (IL)-10 and transforming growth factor-β (TGF-β) and suppress effector T cells, are defective in patients with SLE [19].

The differentiation of CD4^+^ T helper cells into functionally distinct helper T subsets is critical for the pathogenesis of autoimmune diseases [20, 21], especially since the active involvement of T helper (Th) 17 and T follicular helper (Tfh) cells and the dysfunction of T regulatory (Treg) cells have been reported [20, 22–27]. Among these subsets, the Tfh cells have emerged as a critical regulator of autoimmunity [22]. The Tfh cells provide B cell help by promoting the class switching of B cells and are defined by the expression of the master regulator Bcl6 and effector cytokine IL-21, along with key surface molecules, such as PD-1, CXCR5, CD40L, and ICOS [22, 28]. The CXCR5 expression allows Tfh cells to migrate from the T cell zone to the B cell follicle where they localize in the germinal center (GC) and mediate B cell help via cell-cell contact using the co-stimulatory molecules CD40L and ICOS [22]. Thus, B-Tfh cell interaction is necessary for autoantibody production. In mice, the excessive activity of Tfh cells induces hyperactive GC formation and autoantibody production, leading to a SLE-like phenotype [29, 30]. While we and others have reported the mechanism of Tfh differentiation, the exact role of this subset in patients remains elusive. High proportions of circulating Tfh cells, which are characterized as CD4^+^CXCR5^+^ICOS^high^PD-1^high^, have been described in SLE patients, and their level in the peripheral blood correlates with titers of autoantibodies and with disease severity [31, 32].

Taken together, these findings highlight the notion that activated T cells, in addition to activated B cells, may also be potentially involved in the pathogenesis of autoimmunity and that the interaction between activated B and Tfh cells may play an important role in autoantibody-driven autoimmune diseases.

## Pathological role of BAFF and APRIL in autoimmune diseases

**Fig. 1:**
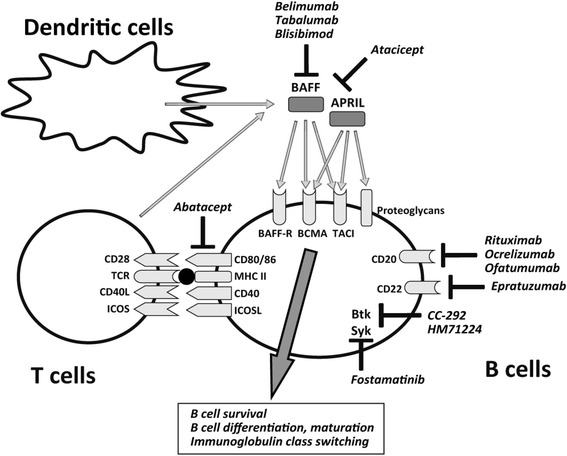
Emerging B cell-targeted therapy including BAFF/APRIL inhibition in autoimmune diseases. Current strategies for autoimmune diseases include appropriate targets for therapeutic modulation such as B cell surface antigens (CD20 and CD22), co-stimulatory molecules (CTLA-4, CD40/CD40L, ICOS/ICOSL, and BAFF/APRIL/BAFF-R/BCMA/TACI), and various intracellular signal transduction pathways (Syk and Btk)

BAFF, also called B lymphocyte stimulator (BLyS), is a B cell activation factor which is mainly expressed by monocytes, macrophages, and activated T cells. BAFF can be expressed on the cell surface as a membrane-bound form or released as a soluble form after cleavage by furin. BAFF binds to three receptors, the BAFF-R, BCMA, or TACI, and regulates B cell survival, differentiation, maturation, immunoglobulin class switching, and antibody production (Fig. 1) [33, 34]. BAFF-R is mainly expressed in immature B cells, whereas TACI and BCMA are expressed in matured memory B cells and plasma cells, respectively. In addition, APRIL, which is a homologous factor to BAFF, binds to TACI, BCMA, and proteoglycans (Fig. 1). APRIL forms heterotrimers with BAFF and enhances BAFF-mediated B cell activation [35]. TACI binds with higher affinity to APRIL but lower affinity to BAFF, compared with other BAFF receptors. Although both BAFF and APRIL promote B cell survival and differentiation, there are complicated regulatory mechanisms according to the varieties of the receptors (BAFF-R, BCMA, or TACI) and the differentiation stage of B cells, as described above. In addition to its effect on B cells, recent works have demonstrated that BAFF can promote T cell activation, proliferation, and differentiation [36]. Interestingly, Coquery et al. reported that BCMA negatively regulates Tfh cell expansion, while BAFF-R-mediated signaling promotes Tfh cell accumulation into GC in lupus-prone mice [37]. Thus, the balance between BCMA and BAFF-R signaling may control the development of Tfh cells, indicating that BAFF/APRIL regulate autoimmunity not only via survival and differentiation of B cell but also via expansion of Tfh cells.

Animal studies have shown that BAFF-deficient mice lack B cell maturation and the knockout of BAFF in lupus-prone mice showed a reduction of mortality and disease severity [38]. The transgenic mice for BAFF show an expanded B cell maturation and develop severe SLE, which is supported by evidence on increased concentrations of anti-double-stranded DNA (dsDNA) antibodies and immune complex deposition in the mesangium [34, 39–41]. In humans, the serum level of BAFF and APRIL is both elevated in patients with SLE and positively correlates with disease activity and serological markers such as anti-dsDNA antibody levels [42, 43]. There is a correlation between BAFF levels and circulating autoantibody levels in Sjogren’s syndrome (SS) [44]. In addition, BAFF has been found to be elevated in the serum of AAV patients [45, 46]. These results suggest a potential therapeutic strategy for patients with systemic autoimmune diseases by BAFF and/or APRIL blockade.

## Targeting BAFF and APRIL in systemic autoimmune diseases

1. BAFF blockers

Current strategies for autoimmune diseases include appropriate targets for therapeutic modulation such as B cell surface antigens (CD20 and CD22), co-stimulatory molecules (CTLA-4, CD40/CD40L, ICOS/ICOSL, and BAFF/APRIL/BAFF-R/BCMA/TACI), and various intracellular signal transduction pathways (Syk and Btk) (Fig. 1) [47, 48]. Selective inhibitors of BAFF and APRIL, which should ameliorate the pathogenesis by inhibiting autoreactive B cell activation and autoantibody production, are in clinical trials for autoimmune diseases (Fig. 1).

Belimumab is a fully human monoclonal antibody that antagonizes BAFF, thus inhibiting B cell survival and differentiation [49]. Belimumab directly reduces activation of naïve and transitional B cells and indirectly inhibits development of IgD^−^CD27^+^ class-switched memory B cells, plasmablasts, and plasma cells. The multicenter, randomized placebo-controlled double-blind phase III trials, BLISS-52 and BLISS-76, were performed to investigate the efficacy of belimumab at 1 or 10 mg/kg compared to placebo in the treatment of active SLE [50–52]. The primary end point was amelioration in SRI (SLE responder index), a composite measurement of SELENA-SLEDAI (Safety of Estrogens in Lupus Erythematosus National Assessment-Systemic Lupus Erythematosus Disease Activity Index), BILAG (British Isles Lupus Assessment Group) score, and physician global assessment. The BLISS-52 trial demonstrated that SRI rates at 52-week posttreatment were 44 %, 51 % (*p* = 0.01), and 58 % (*p* < 0.01) in the placebo, belimumab 1 mg/kg, and belimumab 10 mg/kg groups, respectively, suggesting a significant improvement of disease activity with an increased dose of this drug [51]. Belimumab has greater therapeutic benefit in patients with higher disease activity (SLEDAI ≥10), anti-dsDNA positivity, or low complement [53]. No significant difference between the frequency of serious adverse reactions between the belimumab group and the placebo group was observed. Collectively, these results highlighted the efficacy and tolerability of belimumab as a novel biologic agent for the treatment of SLE, and the FDA approved this drug in 2011. However, the patients with active lupus nephritis were excluded in these trials. Therefore, it would be useful to investigate in future trials to elucidate the efficacy of belimumab in the patients with major organ involvements. Currently, the phase III trials to examine the efficacy and safety of belimumab in active lupus nephritis (NCT01639339) and in SLE patients located in Northeast Asia (NCT01345253) are ongoing.

Furthermore, belimumab is currently undergoing clinical trials in SS and AAV. In the phase II trial in 30 patients with primary SS (BELISS), 60 % of the patients were responders and systemic activity scores measured by the EULAR SS disease activity index (ESSDAI) were significantly improved [54, 55]. Since this is an open-label trial, further randomized controlled trials are warranted. The phase III multicenter, randomized, double-blind study to evaluate the efficacy and safety of belimumab in combination with azathioprine for the maintenance of remission in GPA and MPA (BREVAS) is ongoing (NCT01663623) [56, 57].

Other anti-BAFF agents, tabalumab and blisibimod, are also being assessed in phase III randomized placebo-controlled trials to evaluate their efficacy in SLE. Tabalumab is a monoclonal antibody that neutralizes BAFF in both membrane-bound form and soluble form, whereas belimumab is thought to target the soluble form only. In rheumatoid arthritis (RA), tabalumab showed clinical efficacy in phase II trials in patients with an inadequate response to methotrexate (MTX) [58, 59]. However, the phase III trial demonstrated that tabalumab did not provide the degree of clinical efficacy in moderate-severe RA, taking the MTX observed with other approved biological agents [60]. Based on these findings, the pharmaceutical company discontinued the phase III trial for RA. In addition, the phase III clinical trials for tabalumab in moderate to severe SLE (ILLUMINATE-2) met its primary end point only at higher doses but failed to meet secondary end points [61]. The pharmaceutical company also discontinued the development of this drug for SLE.

Blisibimod is a human “peptibody,” which binds to both cell membrane-expressed and soluble BAFF and antagonizes BAFF, and has recently been evaluated in a phase II clinical trial (PEARL-SC) [62]. In this study, the significant reductions in proteinuria and anti-dsDNA and significant increases in C3 were observed with the blisibimod group. Currently, a phase III study to examine the efficacy and safety of blisibimod in patients with active SLE (NCT01395745) is under way.

Briobacept, a protein containing both IgG and the ligand of BAFF-R, which antagonizes BAFF did not show sufficient efficacy in a phase II trial (ATLAS) (NCT01499355) and was terminated.

2. TACI-Ig: atacicept

Atacicept, a recombinant fusion protein containing both the Fc portion of the human IgG1 and the extracellular domain of TACI [63, 64], binds to APRIL and BAFF and inhibits activation of TACI-mediated signaling. The phase I trial in moderately active SLE showed that atacicept resulted in a 60 % reduction in mature B cells and a 45 % attenuation of immunoglobulin compared to placebo [65]. There were no significant differences in the levels of adverse reactions between atacicept and placebo. However, the phase II clinical trial in patients with active lupus nephritis who are taking steroids and MMF was terminated due to severe infection [66]. Isenberg et al. reported recently the results of a randomized phase II/III trial of atacicept that sought to determine the efficacy and safety of atacicept in the prevention of flares in SLE [67]. The results with a high dose of atacicept were encouraging, but there are severe concerns about the infections. Currently, the phase III clinical trials for atacicept in patients who have no major organ involvements (ADDRESS II) (NCT01972568, NCT02070978) are under way. In Japan, a phase IIb trial in patients with SLE is in progress.

## Conclusions

BAFF and APRIL play a prominent role in the pathogenesis of autoimmune diseases. Indeed, a certain number of patients receive benefit from BAFF/APRIL-blocking therapies. On the other hand, several clinical trials have demonstrated the efficacy of the BAFF/APRIL blockade which showed considerable variability in the response to B cell-targeted therapy. Furthermore, increasing evidence points to substantial heterogeneity in the pathogenesis of autoimmune diseases; thus, B cell-targeted therapy may be ineffective in some patients but effective in others. Therefore, objective markers that can predict the effect of BAFF/APRIL-blocking agents should be valuable to the precision medicine linked clinically and to cost-effective therapy.

## Abbreviations

AAV: ANCA-associated vasculitis
APRIL: a proliferation-inducing ligand
BAFF: B cell-activating factor
BCMA: B cell maturation antigen
BCR: B cell receptor
BILAG: British Isles Lupus Assessment Group
BLyS: B lymphocyte stimulator
Breg: B regulatory
ESSDAI: EULAR SS disease activity index
GC: germinal center
GPA: granulomatosis with polyangitis
IL: interleukin
MPA: microscopic polyangitis
MTX: methotrexate
RA: rheumatoid arthritis
SELENA: Safety of Estrogens in Lupus Erythematosus National Assessment
SLE: systemic lupus erythematosus
SLEDAI: Systemic Lupus Erythematosus Disease Activity Index
SRI: SLE responder index
SS: Sjogren’s syndrome
TACI: transmembrane activator and calcium modulator and cytophilin ligand interactor
Tfh: T follicular helper
TGF: transforming growth factor
TLR: Toll-like receptor
Treg: T regulatory
